# Enantiomerically Pure Tetravalent Neptunium Amidinates: Synthesis and Characterization

**DOI:** 10.1002/chem.202001865

**Published:** 2020-06-25

**Authors:** Sebastian Fichter, Sebastian Kaufmann, Peter Kaden, Tobias S. Brunner, Thorsten Stumpf, Peter W. Roesky, Juliane März

**Affiliations:** ^1^ Institute of Resource Ecology Helmholtz-Zentrum Dresden-Rossendorf Bautzner Landstraße 400 01328 Dresden Germany; ^2^ Institute of Inorganic Chemistry Karlsruhe Institute of Technology Engesserstraße 15 76131 Karlsruhe Germany

**Keywords:** actinides, amidinates, coordination chemistry, neptunium, transuranium chemistry

## Abstract

The synthesis of a tetravalent neptunium amidinate [NpCl((*S*)‐PEBA)_3_] (**1**) ((*S*)‐PEBA=(*S*,*S*)‐*N*,*N′*‐bis‐(1‐phenylethyl)‐benzamidinate) is reported. This complex represents the first structurally characterized enantiopure transuranic compound. Reactivity studies with halide/pseudohalides yielding [NpX((*S*)‐PEBA)_3_] (X=F (**2**), Br (**3**), N_3_ (**4**)) have shown that the chirality‐at‐metal is preserved for all compounds in the solid state. Furthermore, they represent an unprecedented example of a structurally characterized metal–organic Np complex featuring a Np−Br (**3**) bond. In addition, **4** is the only reported tetravalent transuranic azide. All compounds were additionally characterized in solution using *para*‐magnetic NMR spectroscopy showing an expected *C*_3_‐symmetry at low temperatures.

## Graphical abstract

**Enantiopure transuranic complexes**: The first structurally characterized enantiopure complexes of neptunium have been synthesized. Starting from a chloro tris(amidinate) complex unprecedented neptunium(IV) fluoro, ‐bromo and ‐azido compounds could be yielded and characterized in solution and in the solid state (see scheme).

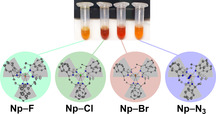

The chemistry of neptunium has always lagged behind that of its lighter neighbor uranium (U)^1^ due to its radiotoxicity and hence small amounts of this man‐made element which must be handled in dedicated research facilities.^2^ Additionally, the lack of available starting compounds confined its use until only recently. In 2014 Reilly et al. published the synthesis of NpCl_4_(dme)_2_ 3 (dme=dimethoxyethane), which enables the synthesis of a number of Np coordination compounds in recent years.^4^ However, many recent studies are limited to one‐step reaction schemes targeting the desired tetravalent Np complex. Additional reactivity studies of the compounds are scarce due to the small amounts of Np starting material available and the precautions needed when handling this material.

Enantiopure complexes of the transuranium (TRU) elements are basically unknown. To the best of our knowledge, no single crystal structure of an enantiopure TRU compound has been reported yet. There is only one publication on Cm complexes ligated by chiral cages; these compounds were characterized in solution and by elemental analysis.^5^

In prior work some of us established a series of rare earth metal complexes ligated by a chiral amidinate.^6^ By using *N*,*N′*‐bis‐(1‐phenylethyl)benzamidinate (PEBA)^7^ as a ligand, a series of mono‐, bis‐, and tris(amidinate) lanthanide complexes were synthesized.6b, 6c All chiral bis‐ and tris(amidinate) complexes have an additional axial chirality. Some of these compounds were investigated as suitable enantioselective catalysts in the hydroamination/ cyclization reactions and the ring opening polymerization of *rac*‐lactide.^8^

Herein, we report the first synthesis of a Np^IV^ complex bearing a chiral ligand, which is also the first example of a structurally characterized enantiopure TRU compound. Additionally, the new complexes also represent the first TRU amidinate compounds, an important class of *N*‐donor ligands, which has already been introduced in main group, transition metal, lanthanide and actinide (Th, U) chemistry.^9^ Furthermore, we were able to functionalize the synthesized compounds by halogen resp. pseudohalogen exchange reactions to yield the first structurally characterized metal‐organic Np complex featuring a Np‐Br bond, as well as an unprecedented Np^IV^ azide.

The Np^IV^ amidinate compound [NpCl((*S*)‐PEBA)_3_] (**1**) was prepared by a salt metathesis reaction using NpCl_4_(dme)_2_ 3 and the lithium salt Li(*S*)‐PEBA6a at ambient conditions (see Scheme 1).

**Scheme 1:**
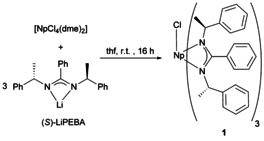
Synthesis of the Np^IV^ amidinate [NpCl((*S*)‐PEBA)_3_] **1**.

The Np^IV^ amidinate **1** was obtained as crystalline solid after workup (see Experimental Procedure in Supporting Information). This chloro complex was used as precursor for several exchange reactions in order to investigate the conformational stability of the enantiopure compound. Thus, **1** was reacted with AgPF_6_, TMSBr and NaN_3_ to give the respective fluoro (**2**), bromo (**3**) and azido (**4**) Np^IV^ trisamidinates (see Scheme 2).

**Scheme 2:**
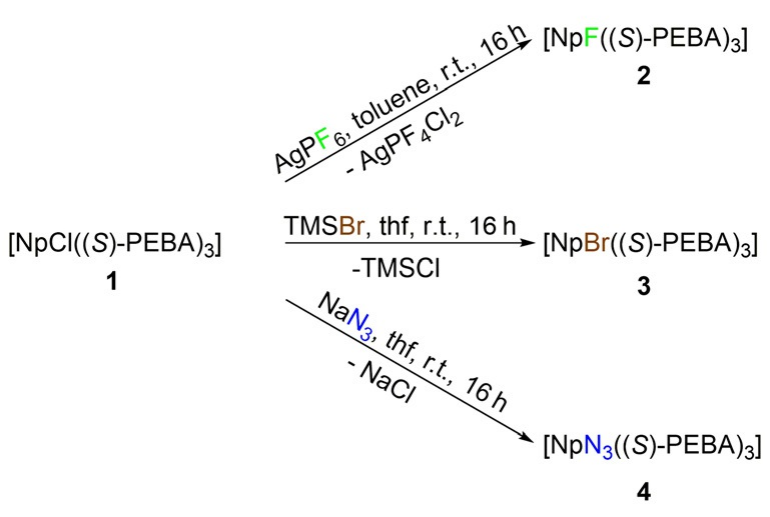
Synthesis of Np^IV^ complexes [NpF((*S*)‐PEBA)_3_] (**2**), [NpBr((*S*)‐PEBA)_3_] (**3**) and [NpN_3_((*S*)‐PEBA_3_] (**4**) from [NpCl((*S*)‐PEBA)_3_] (**1**) by metathesis reactions.

All reactions were carried out under inert conditions yielding the complexes in pure form in high yields and no indication of remaining chloro complex **1** (see NMR spectra in SI). Similar halide substitution reactions have already been reported for thorium and uranium compounds yielding the respective fluoride,^10^ bromide,^11^ and azide10b, 12 compounds. We could show that these reaction conditions are also applicable to Np. The use of AgPF_6_ as a fluorinating agent has been reported by Liddle et al. starting from a U^V^ compound in a redox fluorination.10b However, we applied this reagent in a redox‐neutral approach by starting from the tetravalent Np amidinate **1** to give the tetravalent Np fluoride **2**. The mechanism of the substitution reaction is not fully understood yet (see Supporting Information for more details).

All Np compounds **1**–**4** crystallize in the chiral space group *P*2_1_2_1_2_1_ with similar cell parameters (see Table S7 in Supporting Information) reflecting their isostructurality and even isochirality (see Flack parameters) at the metal center. They exclusively crystallize in the Δ‐configuration.^13^ Note, compound **2** shows deviation of the Flack parameter from 0, indicating some degree of racemization. The molecular structure of **1**–**4** is shown in Figure 1. The Np atom is 7‐fold coordinated by three amidinate ligands and one (pseudo)halide. The amidinate ligands are coordinating asymmetrically to the Np centers represented by a long Np−N_a_ (N_a_=N1, N3, N5; 2.49–2.52 Å) and a short Np−N_b_ (N_b_=N2, N4, N6; 2.36–2.39 Å) bond. The variance between the different compounds is small. Only the Np fluoride **2** possesses slightly longer Np−N bond lengths (max. 3 pm) than compounds **1**, **3**, **4**. This may be caused by the relatively strong Np−F bond thus weakening the Np−N bonds. All Np−N distances are in good agreement with literature data4b, 4g, 14 (see Table S4 in the Supporting Information). Within the amidinate units, the C−N distances are similar in all compounds (ranging from 1.30 Å to 1.40 Å, see Table S3 in Supporting Information) suggesting a delocalization of electron density. Furthermore, the amidinate ligands are tilted against the Np−X bond in such way that a propeller‐like structure is formed with the halide lying on the rotation axis (see Figure 1). Surprisingly, all three ligands possess a different tilting angle (see Table S2 and Figure S9 in the Supporting Information) pointing to only a *C*_1_‐symmetry of the molecules in the solid state. The Np−X distances are growing, as expected, with increasing ion radius of X (Np−F: 2.166(13) Å (**2**), Np−N_3_: 2.23(3) Å (**4**), Np−Cl: 2.630(1) Å (**1**), Np−Br: 2.792(1) Å (**3**)). The Np−Cl distance is in accordance with available literature data,4c, 15 and distances to the halides in **2** and **3** are similar to inorganic Np^IV^ halides^16^ (see Table S5 in the Supporting Information).

**Figure 1:**
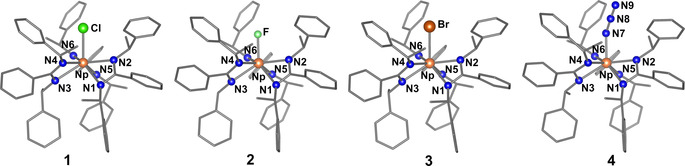
Molecular structures of [NpX((*S*)‐PEBA)_3_] (X=Cl (**1**), F (**2**), Br (**3**), N_3_ (**4**)). Atoms are drawn as spheres. Carbon and hydrogen atoms and solvent molecules are omitted for clarity. Bonds to carbon are shown in gray.

Compounds **1**–**4** were also characterized by infrared spectroscopy in the solid state (see Figure S15). The characteristic C−N stretching vibration of the amidinate unit (1418 cm^−1^) is similar for **1**–**4** indicating also a negligible influence of the halide. The Np−X stretching vibration (X=F, Cl, Br) is anticipated to appear below 650 cm^−1 [17]^ and thus could not be observed with the experimental setup. The only difference between the compounds is the expected intense asymmetric stretching of the azide ion coordinated to the neptunium in **4** (*ν*_as_ (N_3_)=2092 cm^−1^). This is in good agreement with the only other reported Np azido compound ([NpO_2_(N_3_)(Phen)(H_2_O)]_2_ **⋅**3 H_2_O) where an asymmetric stretching frequency of 2090 cm^−1^ is reported.^18^ NMR spectra of the paramagnetic compounds **1**–**4** were recorded at low temperature (243 K) to improve the line width. However, only one set of signals, which is consistent with a *C*_3_‐symmetry of the molecules in solution, is observed. This is another indication of the conformational stability of the chiral Np amidinate ligands in solution. Upon coordination to the Np ion, the symmetry within the ligand is broken. Thus, the number of NMR signals doubles compared to Li(*S*)‐PEBA (see Figure 2). The paramagnetic influence of the Np^IV^ center increases the differences between the chemical shifts of similar nuclei in each set of signals. This is most likely due to a strong distance and angle dependency of a pseudo‐contact contribution of the paramagnetic Np^IV^ center. The paramagnetic influence is best observed in the ^1^H NMR spectra showing a signal range from −11 to 51 ppm. A cutout of the ^1^H NMR spectra of all Np complexes is shown in Figure 2. The complete spectra are depicted in the Supporting Information.

**Figure 2:**
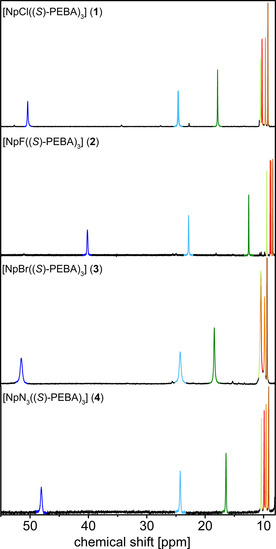
Part of ^1^H NMR spectra of [NpX((*S*)‐PEBA)_3_] (X=Cl (**1**), F (**2**), Br (**3**), N_3_ (**4**)). Similar protons are colored with similar colors (blue/pale blue: NC–H; green/pale‐green: *ortho*‐Ph; red/orange: *meta*‐Ph, brown: *para*‐Ph).

As seen from the spectra, it is clear that the halide ligand has an influence onto the chemical shift of the protons of the attached amidinate ligands. However, this influence is quite small when comparing the spectra of the chloro (**1**), bromo (**3**) and azido (**4**) complexes. By considering that the solid‐state structures of all compounds do not differ substantially from each other and considering that their structures are preserved in solution, the similarities of the ^1^H NMR spectra are not surprising. Interestingly, the fluoride seems to have the strongest influence onto the chemical shifts, as the overall range of signals is significant smaller compared to all other compounds (−6 to 40 ppm). We suggest an electronic influence of the fluoro ligand on the electronic structure of the Np^IV^ ion and hence its paramagnetism as a possible explanation for this observation. Similar effects have already been described for lanthanide and uranium complexes.^19^

Complexes **1**–**4** were additionally characterized in solution using UV/visible/NIR spectroscopy. The spectra are shown in Figure 3. In the region between 300 and 600 nm π–π* transitions of the amidinate ligands are visible. The region between 600 and 1050 nm is dominated by *f*–*f* transitions as already observed by Brown et al. for a Np^IV^ triamidoamine complex.4b A comparison between the spectra of different complexes revealed only minor difference induced by the auxiliary halide ligand. However, subtle differences are again visible for the fluoro compound **2** with an additional transition at 725 nm. This is in good agreement with the observations from NMR spectroscopy, suggesting an electronic influence of the fluoro ligand.

**Figure 3:**
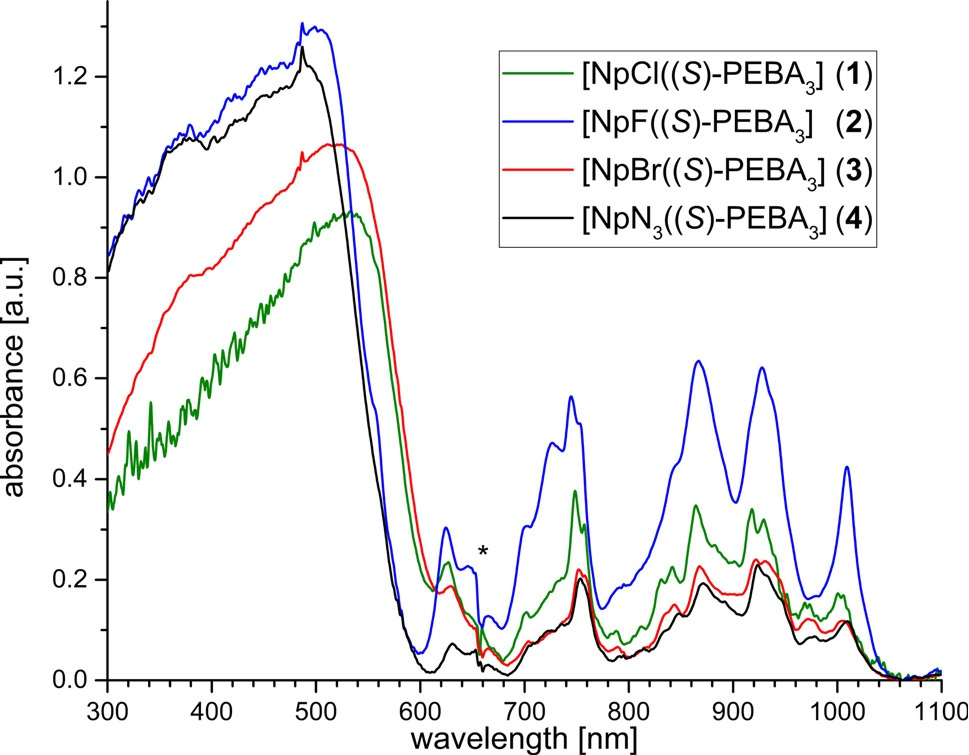
UV/visible/NIR spectra of Np^IV^ complexes **1**–**4** in toluene. Asterisk indicates change of detector.

In summary, a series of isostructural enantiomeric pure tetravalent neptunium amidinates have been presented. They possess the first example of transuranic amidinate molecules and the first structurally characterized chiral TRU complexes. Starting from the newly reported Np chloro complex **1**, halide and pseudohalide exchange reactions are possible. For the first time, this gave access to a structural characterized metal‐organic Np complex having a Np−Br bond. All complexes (**1**–**4**) possess the same chirality‐at‐metal, showing the conformational stability of the complexes upon halide exchange. A comparison of the Np^IV^ complexes in solution revealed the influence of the fluoro ligand onto the electronic structure, potentially altering the crystal field splitting.^20^ This has been comprehensively shown by using paramagnetic NMR and UV/visible spectroscopy. The rich chemistry of the Np^IV^ amidinate system presented, leads us to assume that the (*S*)‐PEBA ligand could serve as suitable spectator ligand for other actinide compounds and would enable further reactivity.

## Conflict of interest

The authors declare no conflict of interest.

## Supporting information

As a service to our authors and readers, this journal provides supporting information supplied by the authors. Such materials are peer reviewed and may be re‐organized for online delivery, but are not copy‐edited or typeset. Technical support issues arising from supporting information (other than missing files) should be addressed to the authors.

Supplementary
