# Supplementary material for: Enantiomerically Pure Tetravalent Neptunium Amidinates: Synthesis and Characterization
Source: Chemistry. 2020 Jun 25;26(41):8867–70. doi: 10.1002/chem.202001865 (PMC7497184; doi:10.1002/chem.202001865)
Supplement: Supplementary file 1 — Supplementary [file CHEM-26-8867-s001.pdf]

Chemistry–A European Journal

Supporting Information

Enantiomerically Pure Tetravalent Neptunium Amidinates: Synthesis and Characterization

Sebastian Fichter,[a] Sebastian Kaufmann,[b] Peter Kaden,[a] Tobias S. Brunner,[b] Thorsten Stumpf,[a] Peter W. Roesky,[b] and Juliane März*[a]

Table of Contents

Experimental 1
General remarks 1
Chemicals 1
Experimental procedure 2
Analytical methods 3
Single-crystal XRD 3
NMR spectroscopy 3
IR spectroscopy 3
UV/visible/NIR spectroscopy 3
Crystal photographs 4
Crystal Structure 4
NMR spectra 9
Fluorine exchange reaction 11
IR spectra 12
Crystallographic data 13
Literature 14
Author Contributions 14

Experimental

Caution! Neptunium (^{237}Np) consists of radioactive nuclides including long-lived α-emitters (^{237}Np; $T_{1/2}$ = 2.14 × 10^6 years). Special precautions as well as appropriate equipment and facilities for radiation protection are required for handling this material. All experiments were carried out in a controlled laboratory at the Institute of Resource Ecology, Helmholtz-Zentrum Dresden-Rossendorf.

General remarks

All preparations were performed under the rigorous exclusion of moisture and oxygen in nitrogen filled glove boxes or using Schlenk techniques. The used solvents were pre-dried over $CaCl_2$ and distilled from Na/K alloy or potassium hydride dispersion and stored over molecular sieve 3 Å prior to use.

Chemicals

The Np starting material $NpCl_4(dme)_2$ (dme = dimethoxyethane) was prepared according to a literature procedure[1]. The lithiated ligand (*S*)-LiPEBA was also prepared according to literature procedures[2]. Ag[PF_6] and NaN_3 (both Sigma Aldrich) were dried for 4 h *in vacuo* to avoid any moisture. All other chemicals were used as received without further purification.

Experimental procedure

[NpCl(S)-PEBA)$_3$]• C_7H_8 (**1**) A 11.1 mg (0.02 mmol, 1 eq.) $NpCl_4(dme)_2$ was dissolved in 1 mL thf. A solution of 19.7 mg (0.06 mmol, 3 eq.) (*S*)-LiPEBA in 1 mL of thf was slowly added resulting in a color change from salmon to dark red. The solution was stirred for 16 h and all volatiles were removed in vacuo yielding a dark red residue. The residue was extracted into 0.5 mL toluene and separated from unsolvable substances by centrifugation. The supernatant was decanted and slowly evaporated yielding single crystals of **1** (18.7 mg, 0.014 mmol, 69%) suitable for structure determination. ^{1}H NMR: (400 MHz, toluene-d$_8$, 243 K) δ[ppm]: 50.47 (s, 1H, NC-H), 24.72 (s, 1H, NC-H), 17.98 (s, 1H, *o*-PhH^{NCN}), 10.62 (s, 1H, *o*-PhH^{NCN}), 10.37 (s, 1H, *m*-PhH^{NCN}), 9.79 (s, 1H, *m*-PhH^{NCN}), 9.37 (s, 1H, *p*-PhH^{NCN}), 6.94 (s, 1H, *p*-PhH), 6.06 (s, 2H, *m*-PhH), 5.97 (s, 3H, Me), 3.45 (s, 1H, *p*-PhH), 2.68 (s, 2H, *o*-PhH), 1.77 (s, 2H, *m*-PhH), -0.47 (s, 3H, Me), -10.94 (s, 2H, *o*-PhH). ^{13}C{^{1}H} NMR: (100.58 MHz, toluene-d$_8$, 243 K): 163.0 (*i*-Ph), 150.3 (*i*-Ph), 133.6 (*o*-Ph^{NCN}, *p*-Ph^{NCN}), 130.4 (*o*-Ph), 129.1 (*m*-Ph), 128.6 (*m*-Ph^{NCN}), 127.1 (*o*-Ph^{NCN}), 125.9 (*p*-Ph), 124.1 (*m*-Ph^{NCN}), 122.7 (*m*-Ph), 120.9 (*p*-Ph), 117.6 (*o*-Ph), 68.1 (NC-H), 53.6 (NC-H), 40.2 (Me), 25.5 (Me), the signals for the N*C*N and *i*-Ph^{NCN} carbons could not be observed. IR (ATR): v [cm^{-1}] 662 (s), 694 (vs), 731 (s), 741 (s), 759 (s), 778 (m), 911 (m), 915 (m), 966 (m), 999 (m), 1010 (m), 1020 (m), 1028 (m), 1081 (s), 1089 (m), 1105 (m), 1129 (m), 1174 (m), 1208 (m), 1275 (m), 1283 (m), 1306 (s), 1327 (m), 1342 (s), 1367 (m), 1372 (m), 1418 (s), 1443 (s), 1491 (m), 2866 (w), 2958 (w). ($C_{76}H_{77}ClN_6Np$, 1346.94 g/mol).

[NpF(S)-PEBA)$_3$]• C_7H_8 (**2**) A solution of 3.2 mg Ag[PF$_6$] (0.013 mmol, 1.1 eq) in 0.25 mL toluene was slowly added to a solution of 15.5 mg **1** (0.012 mmol, 1 eq) in 0.75 mL toluene. The color of the solution changed from dark red to orange. The solution was stirred for 16 h resulting in a turbid orange solution. The solvent was removed *in vacuo* after centrifugation. The dark orange residue was dissolved in 2 mL of pentane to give an orange solution, which was allowed to slowly evaporate under inert conditions to yield **2** as orange crystalline solid (13.1 mg, 0.010 mmol, 82%).^{1}H NMR: (400 MHz, toluene-d$_8$, 243 K) δ[ppm]: 40.15 (s, 1H, NC-H), 22.85 (s, 1H, NC-H), 12.57 (s, 1H, *o*-PhH^{NCN}), 9.49 (s, 1H, *o*-PhH^{NCN}), 8.89 (s, 1H, *m*-PhH^{NCN}), 8.73 (s, 1H, *m*-PhH^{NCN}), 8.45 (s, 1H, *p*-PhH^{NCN}), 6.38 (s, 1H, *p*-PhH), 5.43 (s, 2H, *m*-PhH), 4.57 (s, 1H, *p*-PhH), 3.47 (s, 2H, *m*-PhH), 1.48 (s, 3H, Me), 0.86 (s, 3H, Me), -0.12 (s, 2H, *o*-PhH), -4.76 (s, 2H, *o*-PhH). ^{13}C{^{1}H} NMR: (100.58 MHz, toluene-d$_8$, 243 K): 157.0 (*i*-Ph), 151.5 (*i*-Ph), 131.7 (*p*-Ph^{NCN}), 130.1 (*o*-Ph^{NCN}), 128.6 (*o*-Ph), 127.6 (*m*-Ph) 127.2 (*m*-Ph^{NCN}), 125.3 (*p*-Ph), 124.3 (*m*-Ph), 122.3 (*p*-Ph), 122.2 (*m*-Ph^{NCN}), 121.7 (*o*-Ph^{NCN}), 119.5 (*o*-Ph), 63.7 (NC-H), 54.6 (NC-H), 27.67 (Me), 14.45 (Me), the signals for the N*C*N and *i*-Ph^{NCN} carbons could not be observed. IR (ATR): v [cm^{-1}] 698 (vs), 741 (m), 760 (s), 919 (w), 1010 (w), 1029 (m), 1085 (m), 1112 (m), 1149 (m), 1184 (w), 1208 (m), 1254 (m), 1306 (w), 1345 (w), 1375 (w), 1418 (m), 1449 (m), 1494 (w), 2927 (vw). ($C_{76}H_{77}FN_6Np$, 1330.51 g/mol).

[NpBr(S)-PEBA)$_3$]• C_7H_8 (**3**) A 14 mg (0.011 mmol, 1 eq) [NpCl((S)-PEBA)$_3$] (**1**) was dissolved in 1 mL of thf to yield a dark red solution. Ten drops of TMSBr were slowly added resulting in a color change to salmon. The solutions were stirred for 16 h and the solvents were subsequently removed *in vacuo* to give a dark red oily residue, which was triturated with pentane to yield **3** as a pale red solid (10.6 mg, 0.0076 mmol, 76%). Single crystals of **3** were grown by slow evaporation of a concentrated toluene solution. ^{1}H NMR: (400 MHz, toluene-d$_8$, 243 K) δ[ppm]: 51.43 (s, 1H, NC-H), 24.28 (s, 1H, NC-H), 18.44 (s, 1H, *o*-PhH^{NCN}), 10.51 (s, 2H, *o*-PhH^{NCN}, *m*-PhH^{NCN}), 9.83 (s, 1H, *m*-PhH^{NCN}), 9.42 (s, 1H, *p*-PhH^{NCN}), 6.68 (s, 3H, *m*-PhH, *p*-PhH), 6.44 (s, 3H, Me), 4.28 (s, 2H, *o*-PhH), 3.59 (s, 1H, *p*-PhH), 2.05 (s, 2H, *m*-PhH), -1.40 (s, 3H, Me), -10.41 (s, 2H, *o*-PhH). ^{13}C{^{1}H} NMR: (100.58 MHz, toluene-d$_8$, 243 K): 163.6 (*i*-Ph), 152.3 (*i*-Ph), 133.7 (*o*-Ph^{NCN}, *p*-Ph^{NCN}), 131.3 (*o*-Ph), 129.0 (*m*-Ph), 128.5 (*m*-Ph^{NCN}), 127.1 (*o*-Ph^{NCN}), 126.0 (*p*-Ph), 124.1 (*m*-Ph^{NCN}), 122.9 (*m*-Ph), 121.1 (*p*-Ph), 117.9 (*o*-Ph), 55.2 (NC-H), 53.3 (NC-H), 39.7 (Me), 28.0 (Me), the signals for the N*C*N and *i*-Ph^{NCN} carbons could not be observed. IR (ATR): v [cm^{-1}] 662 (w), 698 (vs), 722 (m), 741 (m), 759 (s), 1029 (w), 1082 (m), 1246 (w), 1306 (w), 1344 (w), 1383 (w), 1418 (m), 1445 (m), 1493 (w), 2924 (w), 2954 (w). ($C_{76}H_{77}BrN_6Np$, 1391.4 g/mol)

[NpN$_3$((S)-PEBA)$_3$]• C_7H_8 (**4**) A solution of 22.2 mg (0.016 mmol, 1 eq.) [NpCl((S)-PEBA)$_3$] (**1**) dissolved in 1 mL of thf was added to 2.4 mg NaN_3 (0.037 mmol, 2.3 eq.). The suspension was stirred for 16 h. All volatiles were removed *in vacuo* and the complex was extracted into 0.5 mL toluene. Vapor diffusion with pentane afforded **4** as crystalline solid (10.1 mg, 0.007 mmol, 47%).^{1}H NMR: (400 MHz, toluene-d$_8$, 243 K) δ[ppm]: 48.10 (s, 1H, NC-H), 24.31 (s, 1H, NC-H), 16.46 (s, 1H, *o*-PhH^{NCN}), 10.41 (s, 1H, *o*-PhH^{NCN}), 9.92 (s, 1H, *m*-PhH^{NCN}), 9.59 (s, 1H, *m*-PhH^{NCN}), 9.15 (s, 1H, *p*-PhH^{NCN}), 6.54 (s, 1H, *p*-PhH), 5.67 (s, 2H, *m*-PhH), 4.24 (s, 3H, Me), 3.67 (s, 1H, *p*-PhH), 2.13 (s, 2H, *m*-PhH), 1.72 (s, 2H, *o*-PhH), 0.26 (s, 3H, Me), -9.56 (s, 2H, *o*-PhH). ^{13}C{^{1}H} NMR: (100.58 MHz, toluene-d$_8$, 243 K): 158.6 (*i*-Ph), 147.7 (*i*-Ph),132.9 (*p*-Ph^{NCN}), 132.2 (*o*-Ph^{NCN}), 131.0 (*o*-Ph), 128.6 (*m*-Ph), 128.0 (*m*-Ph^{NCN}), 125.1 (*p*-Ph), 123.4 (*m*-Ph^{NCN}), 123.2 (*o*-Ph^{NCN}), 122.6 (*m*-Ph), 121.0 (*p*-Ph), 117.3 (*o*-Ph), the signals for the N*C*N, *i*-Ph^{NCN}, N*C*-H and Me carbons could not be observed. IR (ATR): v [cm^{-1}] 663 (s), 698 (vs), 731 (s), 741 (s), 759 (s), 777 (m), 999 (m), 1009 (m), 1019 (m), 1028 (m), 1072 (s), 1089 (m), 1128 (m), 1142 (m), 1176 (m), 1210 (m), 1274 (m), 1283 (m), 1300 (s), 1309 (s), 1325 (m), 1343 (s), 1371 (s), 1420 (s), 1443 (s), 1492 (m), de, 2920 (w). ($C_{76}H_{77}N_9Np$, 1353.53 g/mol)

Analytical methods

Single-crystal XRD

Crystals of all compounds were analyzed on a Bruker D8 Venture single-crystal x-ray diffractometer with micro-focused Mo Kα radiation (λ = 0.71073 Å) and a PHOTON 100 CMOS detector. All the data were collected at 100 K. Single crystals appropriate for the measurement were selected on an optical microscope equipped with a polarization filter, and mounted on a MicroMount^{TM} supplied by MiTeGen, USA, with mineral oil. Generic φ- and ω-scans were performed to collect several sets of narrow data frames. Data treatment was performed with the Bruker APEX 3 program suite including the Bruker SAINT software package for integration[3]. Empirical absorption correction using the Multi-Scan method (SADABS[4]) was applied to the collected data. The structure was solved and refined with full-matrix least-squares data on F^2 using the Bruker SHELXTL[5] software package and the program ShelXle[6]. The tetravalent neptunium amidinate **1** has a Flack parameters (0.014(4)) which is larger than 3σ. However, it is close to zero and hence has not been refined as a two-component twin. The crystals of the neptunium amidinate **2** have all shown signs of non-merohedral twinning. Thus, the crystal used for the structure determination has been integrated as a two component twin. The absorption correction was performed using TWINABS[7]. The structure was solved using the HKLF4 file and finally refined against the HKLF5 file. However, despite the twin refinement the Flack x parameter is too high (0.37(9)) so that an isomerization of the complex molecules could not been ruled out.

NMR spectroscopy

NMR spectra were recorded on a Varian Inova 400 spectrometer with a ^{1}H frequency of 399.89 MHz and a ^{13}C frequency of 100.56 MHz inside a controlled laboratory. Special precautions are taken to avoid contamination. All spectra were recorded with a Varian AutoX ID probe head with z gradient. Deuterated solvents were purchased at Deutero GmbH and dried over potassium mirror prior to use.

IR spectroscopy

IR spectra were measured on an Agilent Cary 630 FT-IR spectrometer equipped with a single-reflection attenuated total reflection (ATR) accessory made of diamond. The measurements were performed in an inert glove box filled with N_2. The spectra were recorded between 4000 and 650 cm^{-1} with a resolution of 2 cm^{-1}.

UV/visible/NIR spectroscopy

UV/visible/NIR spectra were recorded with an J&M Analytik AG TIDAS 100 spectrometer connected with optical fibers to a cuvette housing in the glove box. The spectra were recorded between 200 and 1025 nm with 1 cm cuvettes.

Crystal photographs

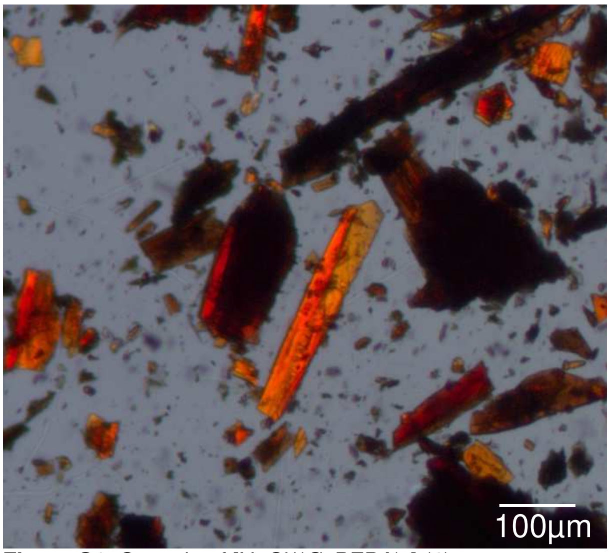

Figure S1. Crystals of [NpCl((*S*)-PEBA)$_3$] (**1**).

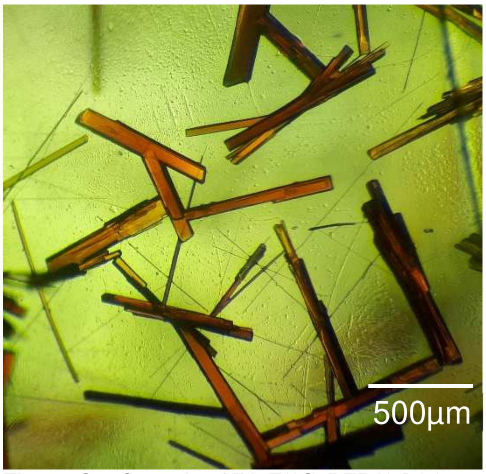

Figure S3. Crystals of [NpF((*S*)-PEBA)$_3$] (**2**).

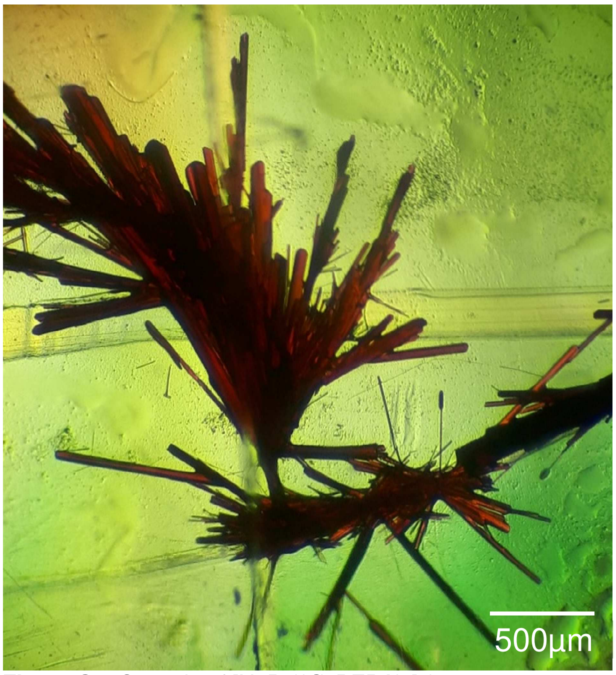

Figure S2. Crystals of [NpBr((*S*)-PEBA)$_3$] (**3**).

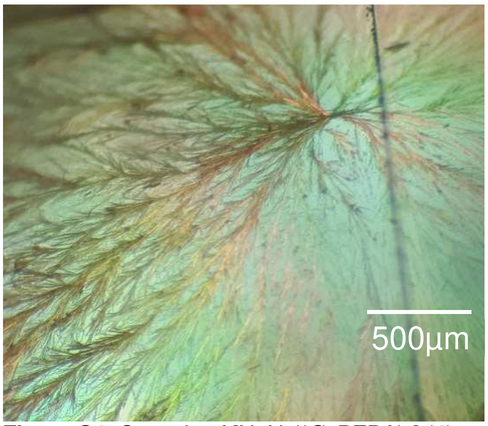

Figure S4. Crystals of [NpN$_3$((*S*)-PEBA)$_3$] (**4**).

Crystal Structure

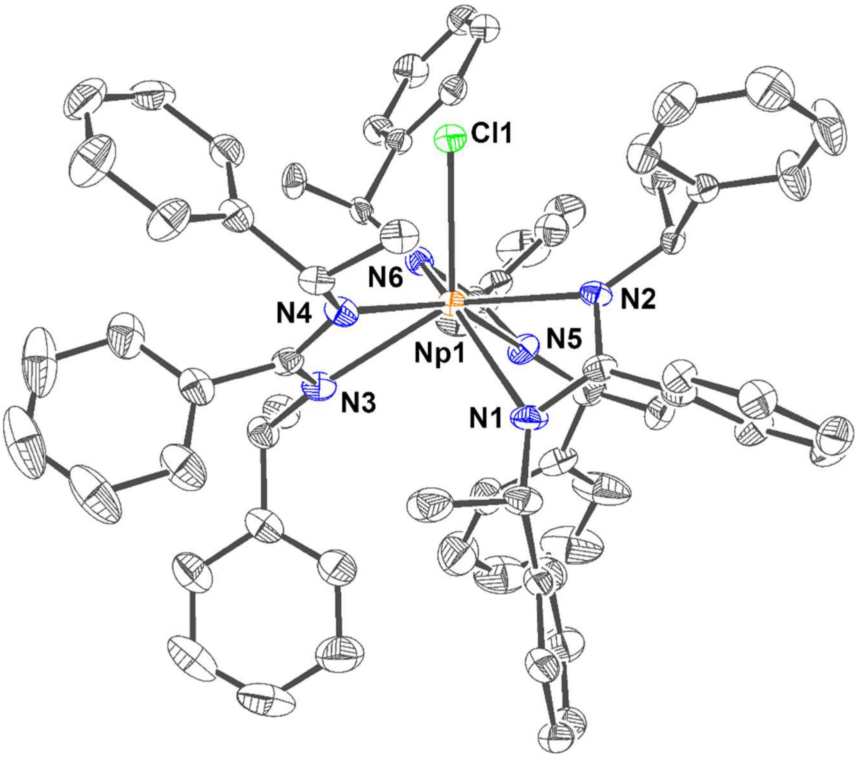

Figure S5. Molecular structure of [NpCl((*S*)-PEBA)$_3$] (**1**). Ellipsoids are drawn at 50 % probability level. Hydrogen atoms and solvent molecules are omitted for clarity. Color code: carbon (C, dark gray), chlorine (Cl, green), nitrogen (N, blue), and neptunium (Np, orange).

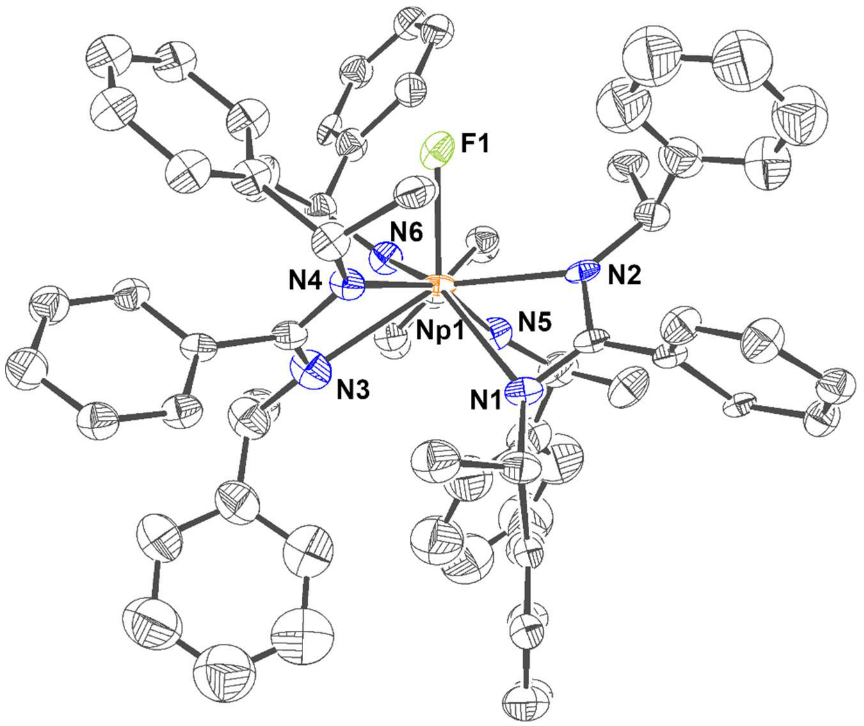

Figure S6. Molecular structure of [NpF((*S*)-PEBA)$_3$] (**2**). Ellipsoids are drawn at 50 % probability level. Hydrogen atoms and solvent molecules are omitted for clarity. Color code: carbon (C, dark gray), fluorine (F, yellow green), nitrogen (N, blue), and neptunium (Np, orange).

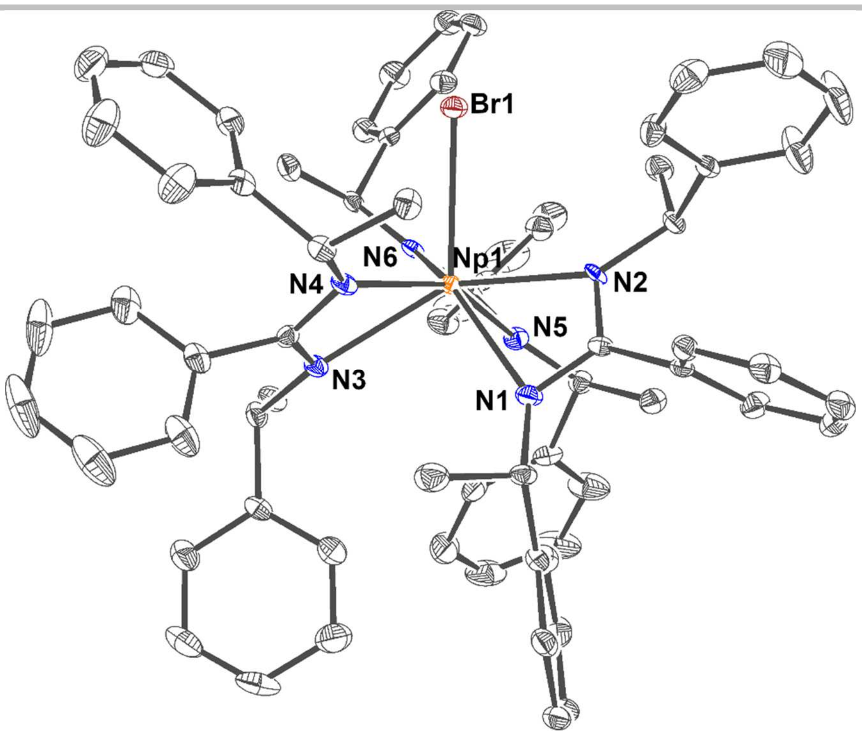

Figure S7. Molecular structure of [NpBr((*S*)-PEBA)$_3$] (**3**). Ellipsoids are drawn at 50 % probability level. Hydrogen atoms and solvent molecules are omitted for clarity. Color code: carbon (C, dark gray), bromine (Br, brown), nitrogen (N, blue), and neptunium (Np, orange).

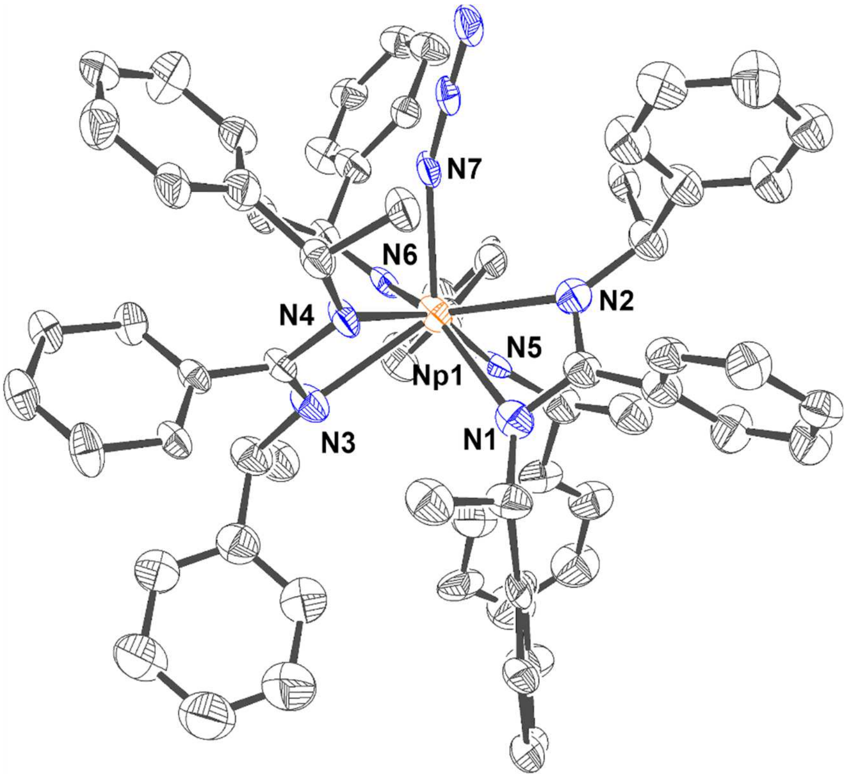

Figure S8. Molecular structure of [NpN$_3$((*S*)-PEBA)$_3$] (4). Ellipsoids are drawn at 50 % probability level. Hydrogen atoms and solvent molecules are omitted for clarity. Color code: carbon (C, dark gray), nitrogen (N, blue), and neptunium (Np, orange).

Table S1. Intramolecular distances between metal center M and coordinating atoms in isostructural [NpX((*S*)-PEBA)$_3$] complexes **1**-**4**

d(M–X) [Å]	**1** (X = Cl)	**2** (X = F)	**3** (X = Br)	**4** (X = N_3)
X	2.630(1)	2.166(13)	2.792(1)	2.23(3)
N1	2.494(5)	2.549(18)	2.488(3)	2.46(3)
N2	2.362(5)	2.430(20)	2.359(3)	2.37(4)
N3	2.494(6)	2.500(20)	2.486(4)	2.47(3)
N4	2.389(5)	2.478(17)	2.382(3)	2.42(3)
N5	2.503(6)	2.530(20)	2.493(4)	2.54(3)
N6	2.353(5)	2.340(20)	2.351(3)	2.36(3)

Table S2. Angles α of plane normals of N1–M1–N2 (A), N3–M1–N4 (B) and N5–M1–N6 (C) against M1–X1 bond. See Figure S9.

	**1** (X = Cl)	**2** (X = F)	**3** (X = Br)	**4** (X = N_3)
A	53.3°	54°	53.8°	57°
B	46.5°	46°	47.1°	43°
C	40.8°	37°	40.4°	38°

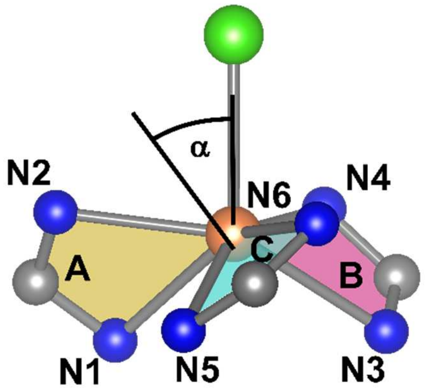

Figure S9. Graphical representation of angle α between plane normal C (N5–M1–N6) against M1–Cl1 bond.

Table S3. Intramolecular distances within amidinate moieties in isostructural [NpX((S)-PEBA)$_3$] complexes **1**-**4**.

d [Å]	**1** (X = Cl)	**2** (X = F)	**3** (X = Br)	**4** (X = N_3)
N1–C9	1.339(8)	1.27(3)	1.337(6)	1.31(5)
N2–C9	1.341(8)	1.37(3)	1.339(5)	1.34(5)
N3–C32	1.349(8)	1.29(4)	1.338(6)	1.31(5)
N4–C32	1.332(9)	1.36(4)	1.337(6)	1.40(5)
N5–C55	1.328(9)	1.37(3)	1.332(6)	1.30(5)
N6–C55	1.332(8)	1.26(3)	1.332(6)	1.39(6)

Table S4. Comparison of intramolecular Np^{IV}–N distances of Np compounds **1**-**4** with literature data of structurally characterized compounds containing Np^{IV}–N bonds (CCDC, 18/11/2019). Bond lengths Np–N1, Np–N3, Np–N5 were averaged to Np–Na and distances Np–N2, Np–N4, Np–N6 to Np–Nb and represent other Np–N distances in cited complexes.

d(Np–X) [Å]	N_a	N_b	N_c
[NpCl((*S*)-PEBA)$_3$] (**1**)	2.497	2.368	
[NpF((*S*)-PEBA)$_3$] (**2**)	2.526	2.416	
[NpBr((*S*)-PEBA)$_3$] (**3)**	2.489	2.364	
[NpN$_3$((*S*)-PEBA)$_3$] (**4**)	2.49	2.38	2.23
[NMe$_4$][Np(SCN)$_8$][8]	2.40		
[Np(H$_2$L^1)$_3$][ClO$_4$][9]	2.497	2.479	
[NpCl$_2$(H$_2$L^1)$_2$][9]	2.454		
[NpCl(L^2)][10]	2.605 (amine)	2.223 (amide)	
[Np(OSiMe$_3$)$_2$(L^3)][11]	2.635 (pyridine)	2.697 (pyrrol)	2.677 (pyrrolidene)

* L^1 = Glutaroimide-dioxime; L^2 = N,N’,N’’-Tris-((tris-isopropyl)silyl)amidotriethylamine (TREN); L^3 =Dipyriamethyrin

Table S5. Comparison of intramolecular Np^{IV}–Hal distances of Np compounds 1-3 with literature data of structurally characterized Np^{IV}–Hal compounds.

d(Np–X) [Å]	F	Cl	Br
[NpCl((*S*)-PEBA)$_3$] (**1**)		2.630	
[NpCl$_4$(DME)$_2$][1]		2.608	
[NpCl$_4$(THF)$_3$][12]		2.586	
[PPh$_4$][NpCl$_6$][13]		2.610	
[NpCl$_3$Cp(Ph$_2$MePO)$_2$][14]		2.641	
[NpF((*S*)-PEBA)$_3$] (**2**)	2.166		
NpF$_4$[15]	2.130, 2.148, 2.198, 2.240, 2.301, 2.303, 2.314, 2.331		
[NpBr((*S*)-PEBA)$_3$] (**3)**			2.792
Cs$_2$NpBr$_6$[16]			2.771

NMR spectra

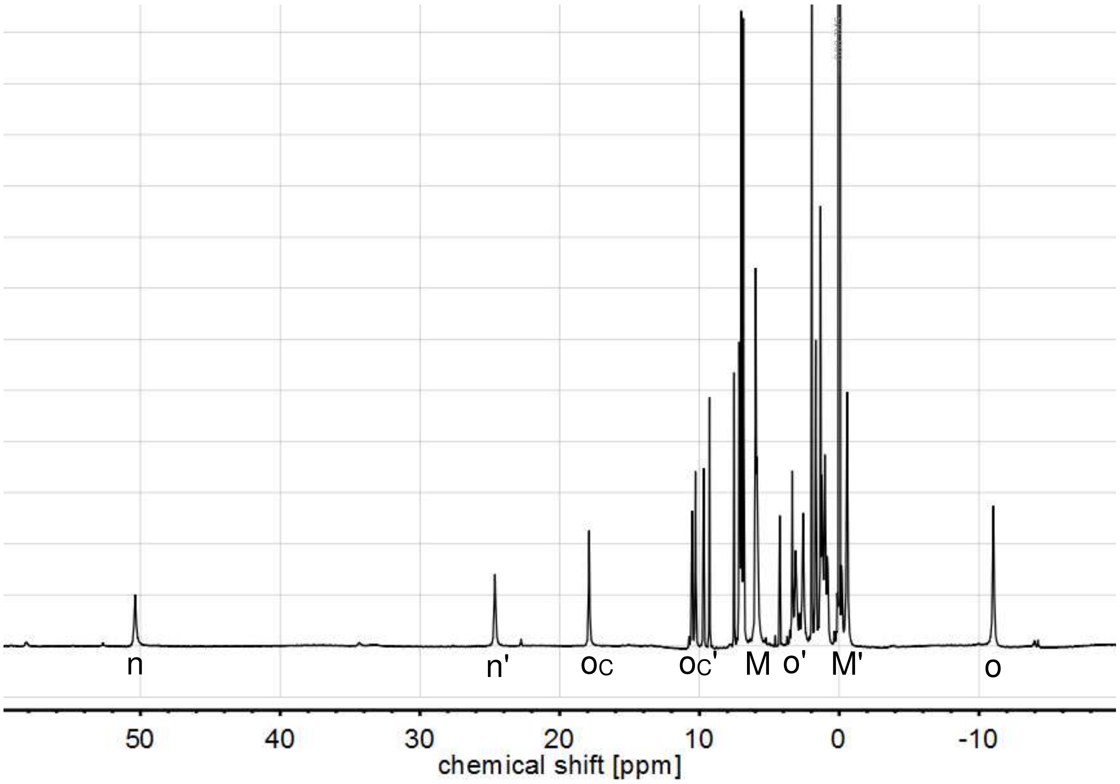

Figure S10. ^{1}H-NMR spectrum of [NpCl((*S*)-PEBA)$_3$] (1) in toluene-d$_8$ at 243 K. Signals: n – NC-H; o – *ortho*-Ph; o$_c$ – *ortho*-Ph (central); M – methyl. Prime indicate protons located on opposite site to auxiliary ligand. Signals of *meta*- and *para*-Ph are not indicated for clarity.

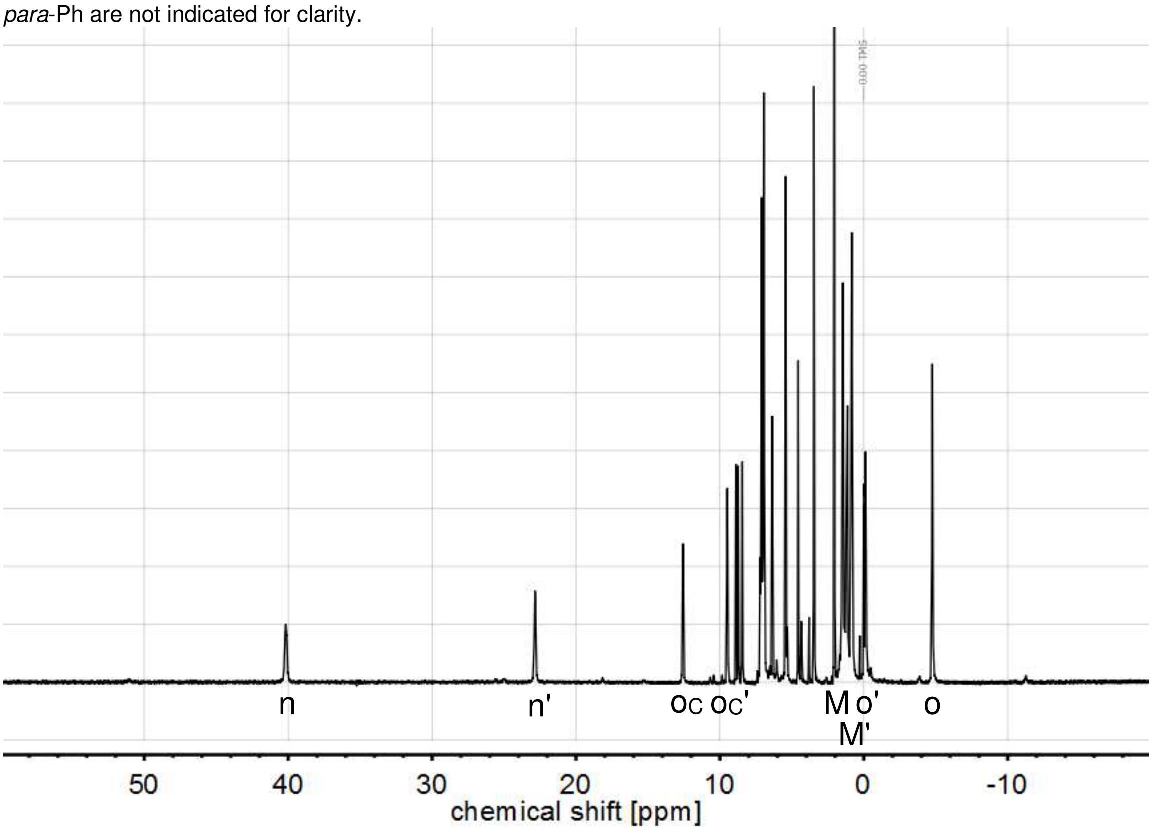

Figure S11. 1H-NMR spectrum of [NpF((*S*)-PEBA)$_3$] (**2**) in toluene-d$_8$ at 243 K. Signals: n – NC-H; o – *ortho*-Ph; o$_c$ – *ortho*-Ph (central); M – methyl. Prime indicate protons located on opposite site to auxiliary ligand. Signals of *meta*- and *para*-Ph are not indicated for clarity.

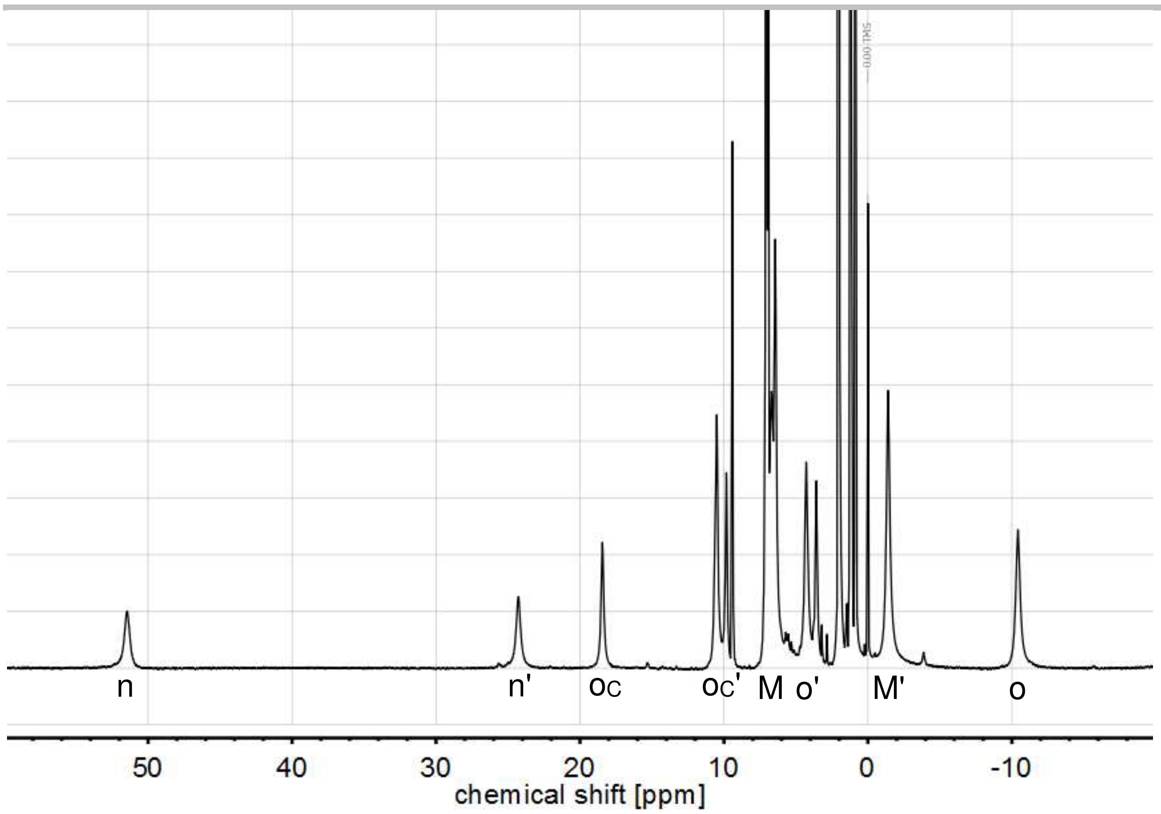

Figure S12. ^{1}H-NMR spectrum of [NpBr(*S*)-PEBA)$_3$] (**3**) in toluene-d$_8$ at 243 K. Signals: n – NC-H; o – *ortho*-Ph; o$_c$ – *ortho*-Ph (central); M – methyl. Prime indicate protons located on opposite site to auxiliary ligand. Signals of *meta*- and *para*-Ph are not indicated for clarity.

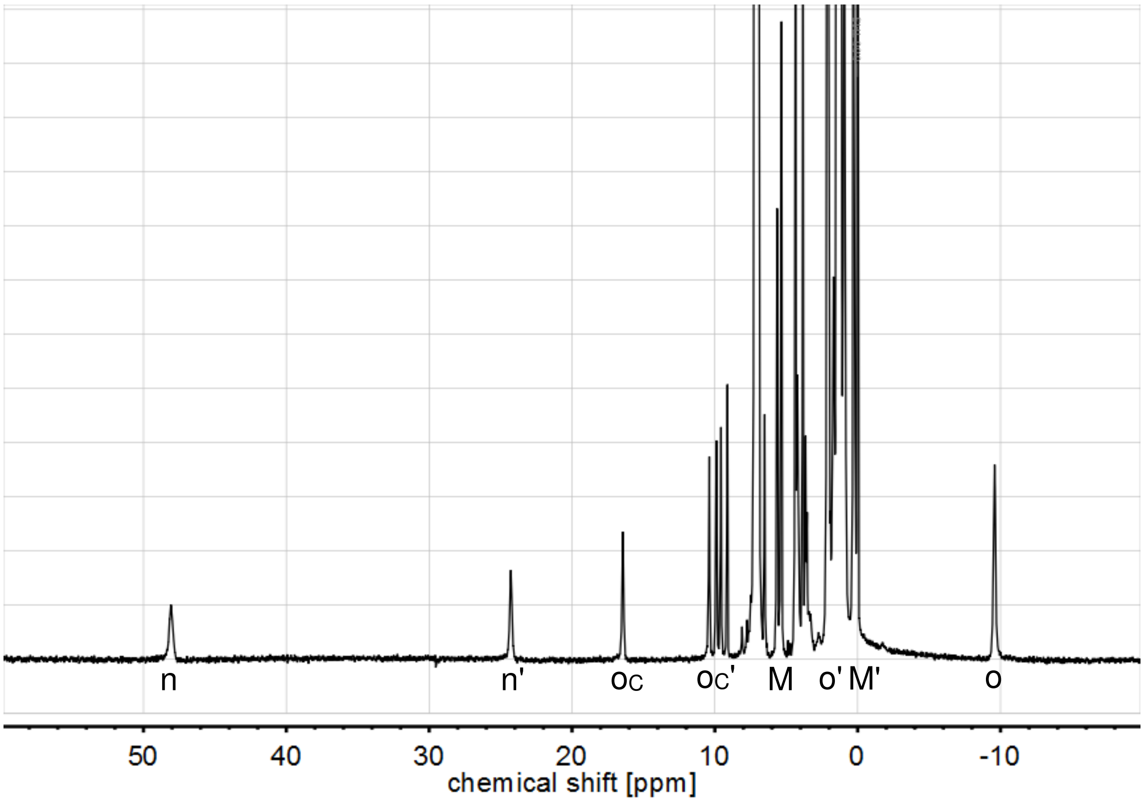

Figure S13. ^{1}H-NMR spectrum of [NpN$_3$((*S*)-PEBA)$_3$] (**4**) in toluene-d$_8$ at 243 K. Signals: n – NC-H; o – *ortho*-Ph; o$_c$ – *ortho*-Ph (central); M – methyl. Prime indicate protons located on opposite site to auxiliary ligand. Signals of *meta*- and *para*-Ph are not indicated for clarity.

Fluorine exchange reaction

To get more insight into the halide exchange reaction according to Scheme 2, ^{19}F NMR spectra of the reaction mixture are recorded (see Figure S14).

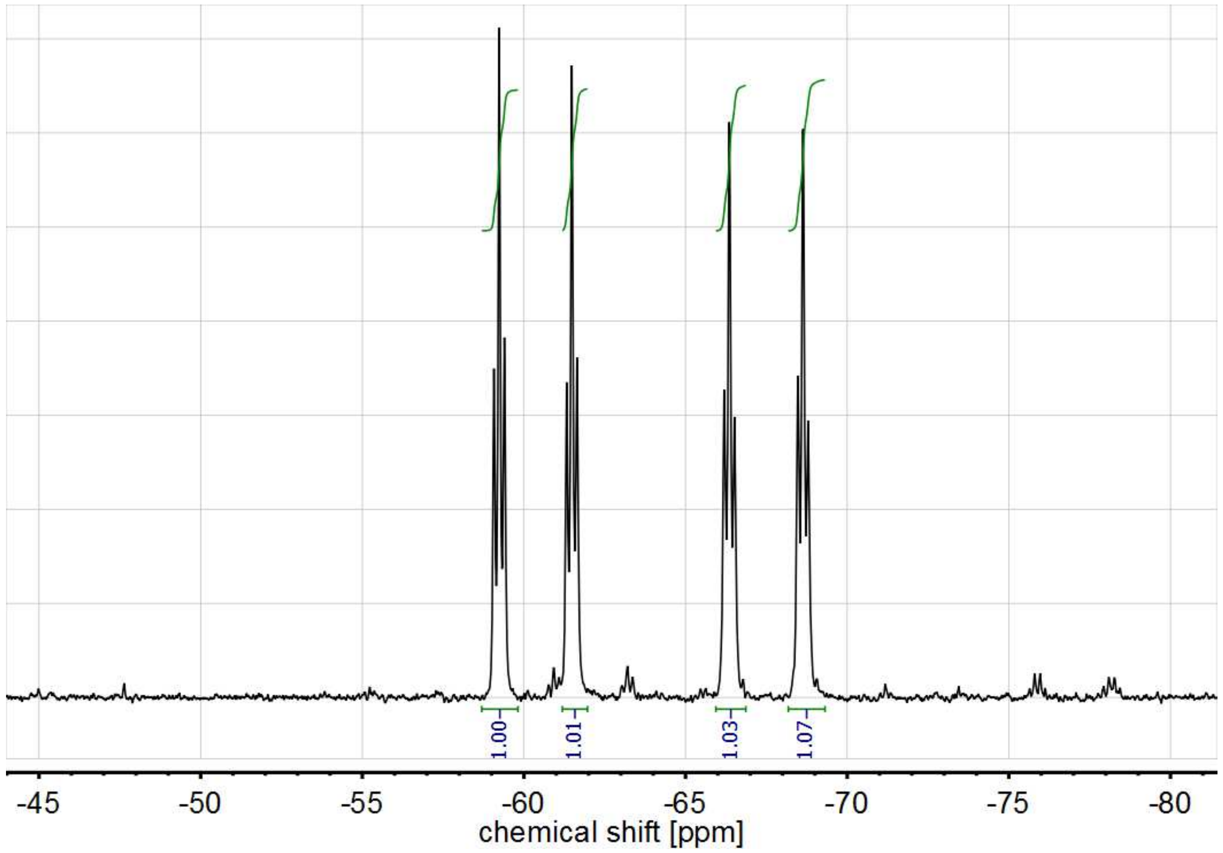

Figure S14. ^{19}F NMR spectra of the reaction mixture of [NpCl((*S*)-PEBA)$_3$] (1) and $AgPF_6$ in toluene-d$_8$ at 243K.

The coupling pattern is in accordance with the formation of a cis-$[PF_4Cl_2]^-$ anion (-60.36 ppm (dt, $^1J_{F\text{-}P}$ = 845.2 Hz, $^2J_{F\text{-}F}$ = 60.9 Hz, 2F, F_{eq}); -67.49 (dt, $^1J_{F\text{-}P}$ = 857.1 Hz, $^2J_{F\text{-}F}$ = 60.5 Hz, 2F, F_{ax}).[17] This is a direct evidence for the halogen exchange reaction between the PF_6^- anion and the neptunium complex **1**. However, no signal of the resulting [NpF((*S*)-PEBA)$_3$] (**2**) was observed. As the fluoride is bound directly to the metal center significant line broadening and paramagnetic relaxation enhancement in addition to the paramagnetic shift could hamper the measurement of the bound fluoride.

The driving force for this reaction is assumed to be of enthalpic nature. This assumption is based on the bond dissociation enthalpies of the involved bonds (see Table S6)

Table S6. Bond dissociation energies D^0 for selected bonds in kJ/mol.

	U-X[18]	Np-X[18]	P-X[19]
Cl	467	n.a.	376
F	659	624	405

Although the values for the Np–Cl bond are not available in the literature the comparison to the uranium and the small difference between the U–F and the Np–F bond let us to consider that the Np–Cl bond dissociation energy is slightly smaller than that for U–Cl. However, the huge difference between the U–F and the U–Cl bond is indicative of the strong Np–F bond, which is formed upon halogen exchange reaction. The energy gain overcomes the energy loss by breaking the P–F bond at the phosphorous.

IR spectra

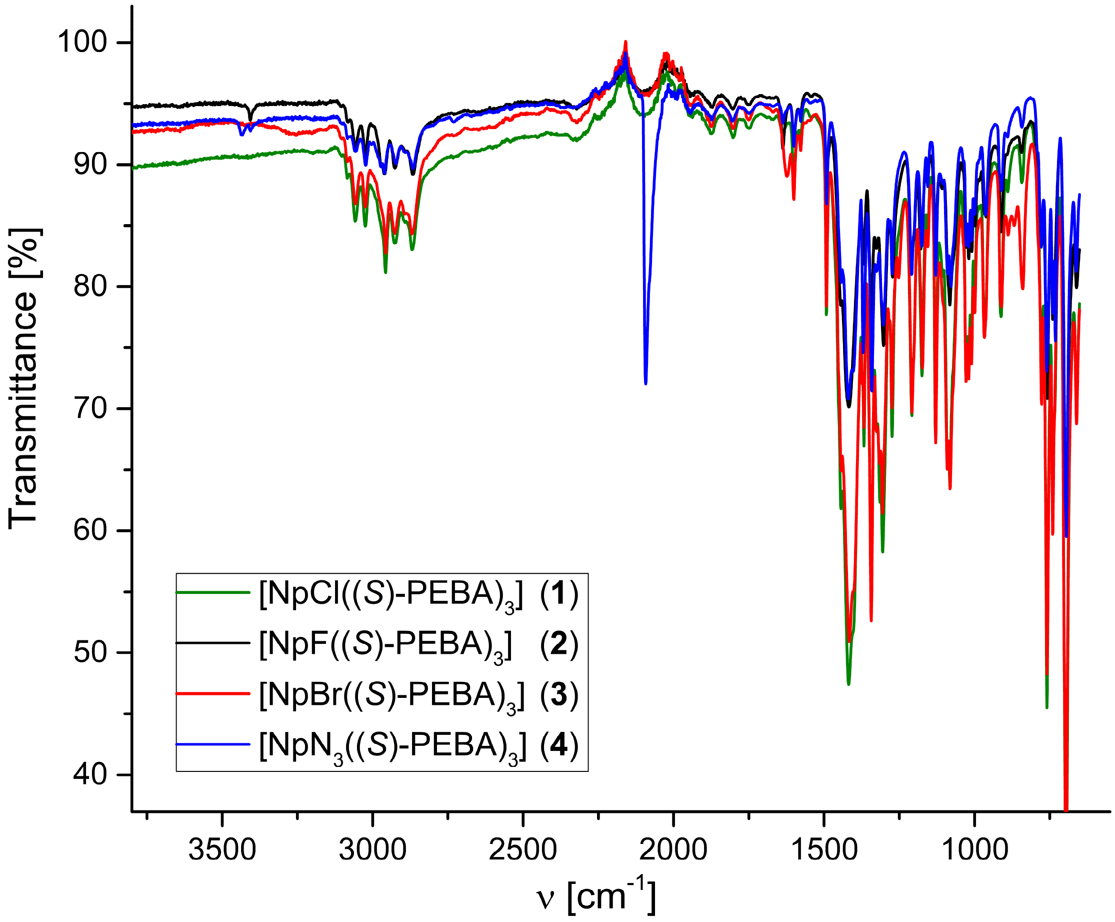

Figure S15. IR spectra of reported Np compounds **1**-**4**.

Crystallographic data

Table S7. Crystallographic data for Np compounds **1**-**4**.

	**1**	**2**	**3**	**4**
Empirical formula	$[C_{69}H_{69}ClN_6Np]\ C_7H_8$	$[C_{69}H_{69}FN_6Np]\ C_7H_8$	$[C_{69}H_{69}BrN_6Np]\ C_7H_8$	$[C_{69}H_{69}N_9Np]\ C_7H_8$
CCDC number	1884774	1978546	1978545	1936874
M (g/mol)	1346.94	1330.43	1391.39	1353.46
Crystal system	Orthorhombic	Orthorhombic	Orthorhombic	Orthorhombic
Space group	$P2_12_12_1$	$P2_12_12_1$	$P2_12_12_1$	$P2_12_12_1$
a (Å)	11.274(2)	11.307(3)	11.3159(7)	11.454(3)
b (Å)	15.356(2)	15.798(4)	15.4137(10)	15.975(4)
c (Å)	36.654(5)	36.118(8)	36.535(2)	36.264(9)
V (Å^3)	6345.8(15)	6452(3)	6372.4(7)	6636(3)
T (K)	100	100	100	100
Z	4	4	4	4
ρ_{calcd} (Mg/m^3)	1.410	1.371	1.450	1.355
abs coeff (mm^{-1})	1.728	1.661	2.306	1.615
Θ_{max} (deg)	25.04	25.05	26.42	19.78
$R\ [I > 2\sigma(I)]^a$	0.031	0.1390	0.025	0.0904
wR2(int)^a	0.101	0.3193	0.051	0.2448
w scheme *d*, *e*	0.0343, 8.9548	0.0002, 591.7440	0.0208, 3.6223	0, 365.3019
Data/Param	11206/752	11412/669	13096/764	5548/631
res. Dens (eÅ^{-3})	1.39, -2.01	2.507, -6.760	1.080, -0.868	3.145/-1.684
R_{int}	0.043	0.085	0.049	0.0914
GooF	1.137	1.149	1.047	1.147
Flack x	0.014(4)	0.37(9)	0.006(4)	0.033(17)

Literature

[1] S. D. Reilly, J. L. Brown, B. L. Scott, A. J. Gaunt, *Dalton Trans.* **2014**, *43*, 1498-1501.
[2] P. Benndorf, C. Preuß, P. W. Roesky, *J. Organomet. Chem.* **2011**, *696*, 1150-1155.
[3] Bruker*, Vol. v2016.9-0*, Bruker AXS Inc., Madison, Wisconsin, USA., **2016**.
[4] G. M. Sheldrick, University of Göttingen, Germany, **1996**.
[5] G. M. Sheldrick, *Acta Cryst.* **2015**, *A71*, 3-8.
[6] C. B. Huebschle, G. M. Sheldrick, B. Dittrich, *J. Appl. Crystallogr.* **2011**, *44*, 1281-1284.
[7] G. M. Sheldrick, Bruker, Madison, Wisconsin, USA, **2012**.
[8] N. A. Budantseva, G. B. Andreev, A. M. Fedoseev, M. Y. Antipin, *Radiochemistry* **2003**, *45*, 335-338.
[9] Z. Zhang, B. F. Parker, T. D. Lohrey, S. J. Teat, J. Arnold, L. Rao, *Dalton Trans.* **2018**, *47*, 8134-8141.
[10] J. L. Brown, A. J. Gaunt, D. M. King, S. T. Liddle, S. D. Reilly, B. L. Scott, A. J. Wooles, *Chem. Commun.* **2016**, *52*, 5428-5431.
[11] J. T. Brewster, D. N. Mangel, A. J. Gaunt, D. P. Saunders, H. Zafar, V. M. Lynch, M. A. Boreen, M. E. Garner, C. A. P. Goodwin, N. S. Settineri, J. Arnold, J. L. Sessler, *J. Am. Chem. Soc.* **2019**.
[12] S. A. Pattenaude, N. H. Anderson, S. C. Bart, A. J. Gaunt, B. L. Scott, *Chem. Commun.* **2018**.
[13] S. G. Minasian, K. S. Boland, R. K. Feller, A. J. Gaunt, S. A. Kozimor, I. May, S. D. Reilly, B. L. Scott, D. K. Shuh, *Inorg. Chem.* **2012**, *51*, 5728-5736.
[14] K. W. Bagnall, G. F. Payne, N. W. Alcock, D. J. Flanders, D. Brown, *J. Chem. Soc., Dalton Trans.* **1986**, 783-787.
[15] W. Zachariasen, *Acta Cryst.* **1949**, *2*, 388-390.
[16] M. Magette, J. Fuger, *Inorg. Nucl. Chem. Lett.* **1977**, *13*, 529-536.
[17] E. G. Il'in, M. N. Shcherbakova, Y. A. Buslaev, *Koord. Khim.* **1975**, *1*, 1179-1189.
[18] P. D. Dau, J. K. Gibson, *J. Phys. Chem. A* **2015**, *119*, 3218-3224.
[19] Y.-R. Luo, *Comprehensive Handbook of Chemical Bond Energies*, **2007**.

Author Contributions

S. F. performed the experiments, analyzed the data and wrote the original draft. S. K. and T. S. B. synthesized the ligands. P. K. measured and analyzed the NMR spectra. J. M. supervised the project and wrote the final draft. T. S. and P. W. R. were responsible for project administration and scientific evaluation.
